# MET in glioma: signaling pathways and targeted therapies

**DOI:** 10.1186/s13046-019-1269-x

**Published:** 2019-06-20

**Authors:** Fangling Cheng, Dongsheng Guo

**Affiliations:** Department of Neurosurgery, Tongji Hospital, Tongji Medical College, Huazhong University of Science and Technology, No.1095, Jiefang Avenue, Wuhan, 430030 China

**Keywords:** Glioma, Glioblastoma, MET, Receptor tyrosine kinase, Targeted therapy

## Abstract

Gliomas represent the most common type of malignant brain tumor, among which, glioblastoma remains a clinical challenge with limited treatment options and dismal prognosis. It has been shown that the dysregulated receptor tyrosine kinase (RTK, including EGFR, MET, PDGFRα, ect.) signaling pathways have pivotal roles in the progression of gliomas, especially glioblastoma. Increasing evidence suggests that expression levels of the RTK MET and its specific stimulatory factors are significantly increased in glioblastomas compared to those in normal brain tissues, whereas some negative regulators are found to be downregulated. Mutations in MET, as well as the dysregulation of other regulators of cross-talk with MET signaling pathways, have also been identified. MET and its ligand hepatocyte growth factor (HGF) play a critical role in the proliferation, survival, migration, invasion, angiogenesis, stem cell characteristics, and therapeutic resistance and recurrence of glioblastomas. Therefore, combined targeted therapy for this pathway and associated molecules could be a novel and attractive strategy for the treatment of human glioblastoma. In this review, we highlight progress made in the understanding of MET signaling in glioma and advances in therapies targeting HGF/MET molecules for glioma patients in recent years, in addition to studies on the expression and mutation status of MET.

## Background

Gliomas comprise the most common type of primary malignant brain tumor, and except for pilocytic astrocytoma and subependymal giant cell astrocytoma, nearly all are characterized by a high recurrence rate, a lack of effective treatment strategies, high rates of mortality, and short survival times. According to the CBTRUS statistical report of the central nervous system tumors in the United States in 2010–2014, gliomas account for approximately 26.6% of all brain tumors, and glioblastoma represents the majority of gliomas (56.1%) and 47.1% of all malignant brain tumors [1]. Only 5.5% of patients typically survive 5 years post-diagnosis and the median overall survival is still dismal at approximately 14.5–16.6 months even with multimodal therapy comprised of surgery, radiotherapy, and chemotherapy [1, 2].

According to the World Health Organization (WHO) classification of tumors of the central nervous system, gliomas can be categorized into four grades (grade I to IV), among which grade IV is also called glioblastoma or glioblastoma multiforme (GBM) [3]. Moreover, a gene expression-based molecular classification of glioblstoma has been presented, including proneural, neural, classical, and mesenchymal subtypes [4]. Despite the identification of these different subtypes, no effective targeted therapy for gliomas has been developed in recent decades to improve outcomes, and most low-grade gliomas (WHO grade I and II) are inevitably recurrent and progress to high-grade gliomas (WHO grade III and IV) [5].

Genetic alterations in glioma occur frequently. Apart from histological classification, genetic diagnoses are recommended to identify the status of isocitrate dehydrogenase 1/2 (*IDH1/2*) mutation, telomerase reverse transcriptase (*TERT*) promoter mutation, 1p/19q co-deletion, BRAF mutation, and O6-methylguanine-DNA methyltransferase (*MGMT*) promoter methylation, which can help to estimate the prognosis and direct treatment options [6]. Nevertheless, to date, effective targeted therapies related to these molecules have not been developed for widespread clinical use and the median overall survival for glioblastomas is still dismal at approximately 14.5–16.6 months [2].

Gene amplifications or mutations are most common among receptor tyrosine kinase (RTK)/RAS/PI3K, p53, and RB signaling pathways, and approximately 86% of glioblastoma samples harbor at least one genetic event in the core RTK/PI3K pathway [7]. RTKs are cell-surface receptors that are activated by ligands, activating mutations, or other mechanisms of dysregulation, all of which contribute to the malignancy of many solid tumors such as non-small cell lung cancer, breast cancer, gastric cancer, hepatocellular carcinoma, and glioblastoma [8–12]. Certain targeted therapies for RTK pathways have significant efficacy for many solid tumors such as breast cancer and lung cancer, but targeted therapies for epidermal growth factor receptor (EGFR) and vascular endothelial growth factor (VEGF) in glioblastoma often result in resistance due to activation of the MET signaling pathway [13–16]. An increasing number of studies have demonstrated that the mesenchymal-epithelial transition factor (MET) and its ligand hepatocyte growth factor (HGF) play a critical role in the proliferation, survival, migration, invasion, angiogenesis, stem cell characteristics, and therapeutic resistance and recurrence of glioblastomas [15–20]. Here, we review the current understanding of MET signaling in gliomas and associated targeted therapies based on preclinical and clinical studies, which provide hope for combined targeted treatment strategies, exploiting this pathway, in the future.

## General mechanisms of glioma

Gliomas are the most lethal primary brain tumors, among which glioblastoma is characterized by a high rate of angiogenesis and aggressive invasiveness, and is resistant to all current therapeutic options. A mechanistic understanding of glioma initiation and progression is complicated by the complexity of genetic and environmental initiating events and the lack of clarity regarding the original cell or tissue. Gene mutations seem to be the most important and well-studied mechanism underlying the formation of gliomas.

The tumor suppressor gene TP53, p16, and phosphatase and tensin homolog (PTEN) phosphatase control cell cycle progression and proliferation, the mutations in or loss of these tumor suppressor genes contribute to the initiation or formation of gliomas [12], and have been demonstrated to be characteristics of many glioblastoma cell lines [21]. The genes encoding IDH1, and to a lesser extent IDH2, were found to be mutated in lower grade gliomas and a subset of glioblastomas that evolved from lower grade tumors, which results in the decreased production of α-ketoglutarate (α-KG) from isocitrate and also the conversion of α-KG to 2-hydroxyglutarate (2-HG) [22]. These changes in metabolites induce extensive DNA hypermethylation by suppressing the function of the ten-eleven translocation (TET) protein [23, 24]. Even through IDH mutations were found to occur earlier than TP53 mutations in low-grade gliomas [25], the underlying mechanism of this phenomenon is still unclear. Despite the fact that *MGMT* (O6-methylguanine-DNA methyltransferase) promoter methylation results in its transcriptional silencing and increases chemosensitivity to temozolomide (TMZ) [26], the dismal prognosis associated with many primary glioblastomas without *MGMT* promoter methylation still has not changed with current therapies. Moreover, *TERT* promoter mutations (C228T, C250T) were found to be associated with significantly shorter progression-free survival (PFS) and overall survival (OS) time in grade III and IV glioma patients [27]. Another mutation is the loss of ATRX (α-thalassemia/mental retardation syndrome X-linked gene), which promotes tumor growth and impairs nonhomologous end joining DNA repair in glioma [28]. All of these gene variations illustrate the possible mechanisms underlying glioma initiation or formation. However, in clinical practice, effective therapy targeting these variations after surgery have not emerged.

Although receptor tyrosine kinases (RTKs) possess the roles as key regulators of normal cellular processes, the dysregulation of growth factor signaling pathways via amplification and the mutational activation of receptor tyrosine kinase (RTK)-encoding genes has been identified as important events in human glioblastomas, and approximately 86% harbor at least one genetic event in the core RTK/PI3K pathway [7]. The amplification and activation of EGFR, platelet derived growth factor receptor α (PDGFRα), and mesenchymal-epithelial transition factor (MET) are the top three desregulated RTKs, which promote the proliferation and invasion of glioma cells [29]. Modern targeted therapies that inhibit RTKs or their ligands have shown promising anti-cancer activities (e.g gefitinib for lung cancer and bevacizumab for colorectal cancer) in other diseases, but their efficacy for glioblastoma has been limited in clinical practice [12, 13, 30]. Further, MET activation is associated with resistance to EGFR- and VEGF-targeted therapy [15, 16], and therefore, this pathway plays an important role in the formation and progression of gliomas. For these reasons, a thorough understanding of MET signaling in glioma, which has been sought in recent years, should be a priority, and perhaps new treatment strategies will emerge in the near future.

## Expression of MET and HGF in glioma

The human *MET* proto-oncogene is located on chromosome 7q31 and *HGF* is located on chromosome 7q21.1 [31]. Emerging lines of evidence have demonstrated that MET is involved in crucial parts of glioma cell biology like tumor proliferation, growth, migration, invasion, and angiogenesis, as well as stemness [17–19]. Earlier analyses of TCGA data showed that approximately 30% of glioblastomas display the overexpression of HGF and MET, suggesting that autocrine HGF activation can occur in the patient population [32]. Moreover, MET was identified in the cytoplasm and at the cell membrane based on immunohistochemical staining, and strong MET expression was found in tumor cells, blood vessels, and peri-necrotic areas of glioma samples, with high MET intensity correlating with high WHO grade and shorter PFS and OS in patients with glioblastoma [33–35].

One study searched for genetic alterations in glioblastomas occurring with or without IDH1 mutations (typical for secondary and primary glioblastoma) using data from The Cancer Genome Atlas (TCGA) and identified 25 genes, of which 21 were located at 7q31–34 [36]. Further analysis of the *MET* gene at 7q31.2 showed that gain occurred in 47% of primary and 44% of secondary glioblastomas [36], suggesting that this genetic alteration plays a role in the pathogenesis of both glioblastoma subtypes. Moreover, activating mutations in MET are significant events during the progression of low-grade gliomas to secondary glioblastomas [20]. Further, MET gain in diffuse astrocytomas was found to be associated with shorter OS time (median, 43.0 vs. 70.7 months; *p* = 0.004) [36]. However, based on IHC staining, contradictory results have been noted; specifically, high MET intensity was not found to correlate with survival for patients with WHO grade II gliomas [33]. In glioblastoma, the overexpression of MET with predominant weak-to-moderate staining intensity was observed in 23% of unamplified glioblastomas, and only strong immunostaining was suggested to be appropriate for the assessment of MET amplification [37], which might also suggest other mechanisms of MET overexpression.

Apart from autocrine HGF secretion, paracrine HGF secretion from neurons and the vasculature facilitates glioma invasion and augments the chemotactic invasion and proliferation of cells that are MET-positive [38, 39]. Further, HGF can act as a chemokine for microglia and might be responsible for their infiltration in malignant gliomas [40]. All of these mechanisms could facilitate the aggressive progression of glioblastoma.

## MET amplification and activating mutations in glioma

To delineate the functions of MET in glioma, it is of primary importance to understand mutations in the MET signaling pathway. One animal study showed that MET amplification is one of the most significant oncogenic events in transgenic mouse models of glioblastoma formation [41]. Moreover, in clinical specimens, 4% of glioblastomas harbor an amplification in MET resulting in the overexpression and constitutive activation of this kinase [7]. The auto-activating METΔ7–8 mutation represents a novel variant of MET, with a deletion in exon 7 and 8, which was detected in 6% of high-grade gliomas [42]. Fusion transcripts of the *MET* gene comprise another activating mutation. These include PTPRZ1-MET (ZM), which was revealed in an RNA-seq study of 272 gliomas conducted by Bao et al. [43], and the previously unknown TFG-MET and CLIP2-MET fusions, which were detected among pediatric glioblastomas in the International Cancer Genome Consortium PedBrain Tumor Project [44]. These MET fusions and activating mutations upregulate mitogen-activated protein kinase (MAPK) signaling, and in cooperation with compromised cell cycle regulation, induce the formation of aggressive glial tumors in vivo [42, 44].

MET overexpression, amplification, and mutation events, based on recent studies, are summarized in Table 1, along with associated methodologies. It would also appear that the detection of MET amplification in glioblastoma depends on both the technique used and the proportion of amplified cells in the tumor. For example, fluorescence in situ hybridization (FISH) is considered more sensitive than comparative genomic hybridization (CGH)-array for the detection of focal MET amplification [37]. Moreover, qPCR and Sanger sequencing have yielded some differences in results. Notably, different antibodies that recognize various MET epitopes and domains have also resulted in diverse staining intensities by IHC. Despite these differences, the results have revealed obvious variations in MET in glioblastoma.

**Table 1:** Molecular alterations of MET in human gliomas

Alteration	Findings	Population	Technique	Evaluation	Ref.
Overexpression	31.2% (63/202) of GBMs displayed overexpression of MET.	TCGA data	CGH	Analyzed TCGA Network datasets from 202 patients via in silico assays for the expression of MET.	[32]
Overexpression	45% (31/69) of glioblastoma patients displayed positive expression of MET.	Turkey	IHC	Tumors were scored positive if more than 30% of cells expressed c-Met.	[34]
Overexpression	79% (15/19) of the patients with recurrent GBM displayed MET overexpression. 37%(7/19) of the patients with primary GBM displayed MET overexpression.	China	IHC	Tumors were scored positive if more than 30% of cells expressed c-Met.	[35]
Amplifcation	MET gain was detected in primary glioblastomas (16/34, 47%) and secondary glioblastomas (16/36, 44%). MET gain was also common in diffuse astrocytomas (43/112, 38%), but less frequent in oligodendrogliomas (13/82, 16%).	Switzerland, Germany, Japan, France	qPCR	Gain was considered as a copy number > 2.699.	[36]
Mutation and fusion genes	The frequency of METex14 in secondary GBM is 14% (11/78), in LGG is 1% (6/530) and in primary GBM is 1.7% (3/174). ZM fusions were identified in four secondary GBM cases co-occur with METex14.	China, Korea	Sanger sequencing	Certain primers and DNA polymerase were used to amplify the fragments. The amplification product bands were extracted from agarose gel after electrophoresis and verified by Sanger sequencing with normal sequence.	[20]
Amplification	MET amplification was detected in four cases in a cohort of 108 GBM.	France	CGH, FISH	For CGH, MET amplification was defined by a log ratio cya5/cya3 > 1.8. For FISH, amplification of MET was defined as more than six copies of MET gene per cell and a ratio MET/CEN7 > 2.2 in more than 10% of cells.	[37]
Overexpression	MET overexpression (> 10%) was detected in 27 out of 104 nonamplified GBM.	France	IHC	The percentage of positive cells > 10% was considered as MET overexpressed.	[37]
Amplification	4% of GBM harbor an amplification of MET gene.	TCGA data	Sanger sequencing	Whole-genome-amplified genomic DNA samples from tumours and normal samples were sequenced by the Sanger method.	[7]
Mutation	METΔ7–8 mutation (lacks exons 7 and 8) is expressed in 6% (6/102) of grade III and IV gliomas.	Netherland	PCR	Performed the exon 6–9 (MET) PCR on cDNA, and then verified by Sanger sequencing.	[42]
Fusion genes	ZM fusion was found in 15% (6/40) of secondary glioblastomas.	China	Sanger sequencing	Two algorithms, deFuse (deFuse-0.6.1) (McPherson et al.2011) and TopHat-Fusion (TopHatFusion-0.1.0) (Kim and Salzberg 2011), were used to detect gene fusion based on the paired-end reads in different samples.	[43]
Fusion genes	Detected two previously unknown fusions of MET:TFG-MET and CLIP2-MET (lack tyrosine 1003 [Y1003], which negatively regulates MET by recruiting ubiquitin ligases), and identified two with a PTPRZ1-MET fusion in 53 pediatric glioblastomas.	German	PCR, DNA sequencing	Paired-end library preparation was conducted using Illumina v2 protocols. Genomic DNA (~ 1 μg) was fragmented to an insert size of ~ 300 bp with a Covaris device, and size selection was performed using agarose gel excision. Deep sequencing was carried out with Illumina HiSeq 2000 instruments.	[44]
Amplification	2% of the 206 GBM cases showed MET amplification.	TCGA data	FISH	In cases where minimum of 1000 tumor cells were present, populations with and without amplification were quantified.	[29]
Mutation	A GGA to GTA mutation, resulting in glycine to valine substitution in codon 1137 of MET was confirmed in one case in all the 11 GBMs.	American	PCR-SSCP	Exons 15, 16, 17, 18, and 19, the most commonly affected regions of the MET gene, was analyzed for MET mutations via SSCP and sequencing.	[105]
Amplification	One glioma (1/11) showed MET amplification exhibiting 20 to 100 copies of MET signal in each affected cell.	American	FISH	At least 100 interphases with strong hybridization signals were scored. Normal brain tissue control showed,6% of cells with one MET gene signal. Alterations of MET copy numbers were scored when present in at least 30% of cells.	[105]
Overexpression	13.1% (18/137) of the GBMs displayed c-Met overexpression.	Korea	IHC	Positivity was measured by Aperio membrane algorithm after scanning with Aperio Scanscope, which appeared as positive %.	[106]
Amplification	5.1% (7/137) of the GBMs displayed MET gene amplifcation.	Korea	FISH	The processing and analysis of the FISH studies were conducted. The signals on 100 non-overlapping intact nuclei were counted.	[106]

## Activation sites of MET

**Fig. 1:**
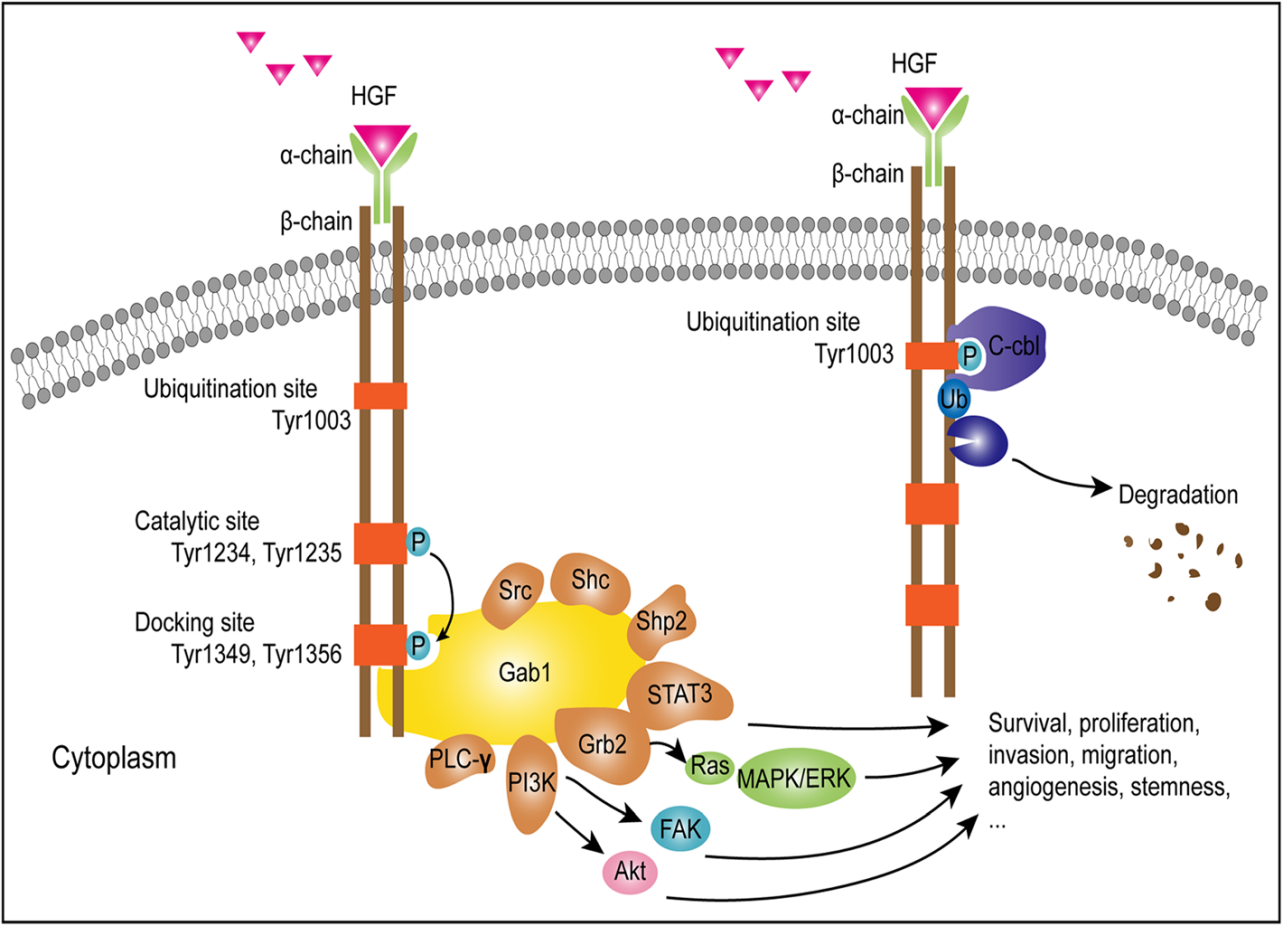
Activation and phosphorylation sites of MET and downstream effects. The activation of MET results in the autophosphorylation of Tyr1234 and Tyr1235 at the catalytic site, and then leads to the subsequent phosphorylation of tyrosine residues Tyr1349 and Tyr1356 in the docking site. The adapter proteins and substrate kinases are recruited and activated (Gab1: Grb2-associated adaptor protein 1; Grb2: growth factor receptor-bound protein 2; Shp2: Src homology protein tyrosine phosphatase 2; Shc: Src homology domain c-terminal adaptor homolog; PLC-γ: phospholipase c-γ; STAT3: signal transducer and activator of transcription 3; PI3K: phosphatidylinositol 3-kinase; FAK: focal adhesion kinase), which facilitates the progression of gliomas. The phosphorylation of MET at cytoplasmic Tyr1003, induces the phosphorylation of c-Cbl, which has intrinsic E3 ubiquitin-protein ligase activity, leading to the degradation and polyubiquitination of MET

MET is a high affinity tyrosine kinase receptor for HGF and consists of α and β subunits. The α-subunit and the amino-terminal region of the β-subunit form the extracellular domain. The remainder of the β-chain spans the plasma membrane and contains a cytoplasmic region with tyrosine kinase activity [45]. The interaction between MET and HGF results in auto-phosphorylation at multiple tyrosine residues, which leads to the recruitment and activation of several signaling effectors including Gab1, Grb2, Src, Shc, Shp2, PLC-γ, FAK, and c-Cbl, as well as the subsequent phosphorylation of downstream transducers such as STAT3, Ras/MAPK/ERK, and PI3K/Akt [46]. Several phosphorylation sites have been studied and are presented in Fig. 1, and their functions are as follows. Phosphorylation events at Tyr1349 and Tyr1356 of the MET kinase domain, which serves as docking sites for intracellular adaptor proteins, are associated with the survival, proliferation, invasion, migration, angiogenesis, and stemness of gliomas [31]. Further, the addition of a phosphate to cytoplasmic Tyr1003 is essential for MET protein ubiquitination and degradation mediated by c-Cbl [20, 47]. Accordingly MET-exon 14-skipping (METex14) results in the omission of exon 14 and the Tyr1003-encoding residue from the MET transcript, which ultimately contributes to prolonged MET stability and constitutive activation [20]. Moreover, the phosphorylation at Tyr1234/1235 within the activation loop of the kinase domain is critical for the subsequent phosphorylation of tyrosine residues Tyr1349 and Tyr1356 near the -COOH terminus [45]. Therefore, phosphorylation status is critical for the controlled regulation of MET activity, which might be of importance for targeted therapy.

## HAI-2 and the HGF/MET signaling pathway in glioma

Regarding dysregulation of the HGF/MET signaling pathway, the *SPINT2* gene has been extensively studied in gliomas. It encodes hepatocyte growth factor activator inhibitor type 2 (HAI-2), which is a membrane-anchored protein and a serine proteinase inhibitor that hinders proteases involved in the activation of HGF [48]. In human gliomas, HAI-2 expression levels are inversely correlated with histological grade, and reduced expression was found to be associated with progression [49]. Moreover, in high-grade glioma, higher SPINT2 expression was determined to be associated with better OS [48]. Basic experimental research also showed that MET phosphorylation levels and glioblastoma tumor growth are reduced by the expression of HAI-2 both in vitro and in intracranial xenografts in nude mice, and that HAI-2 suppresses fibrinolytic activities and inhibits the Matrigel invasion of glioblastoma cell lines [48, 49]. Therefore, these results implied that the downregulation of HAI-2 expression contributes to the progression of glioblastoma through activation of the MET signaling pathway.

## RNA regulation

MicroRNAs (miRNAs) are small non-coding RNAs (containing approximately 22 nucleotides) that function in RNA silencing and the post-transcriptional regulation of gene expression; they can thus regulate oncogenes/tumor suppressors and their associated signal transduction pathways at the cellular level [50]. Long noncoding RNAs (lncRNAs) are more than 200 nucleotides in length and have been shown to play key roles in imprinting control, cell differentiation, immune responses, human diseases, tumorigenesis, and other biological processes [51]. Previous studies have shown that both RNA molecules can affect MET expression or MET signaling pathways in glioblastoma.

MiR-34a, miR-182, and miR-144-3p levels are inversely correlated with MET levels in human gliomas and mechanistic studies have illustrated that they can specifically bind the *MET* 3′-untranslated region and inhibit its expression, thus potently repressing glioblastoma cell proliferation and invasion in vitro and in vivo [52–54]. In contrast, the lncRNA NEAT1 promotes glioma pathogenesis by regulating the miR-449b-5p/MET axis [51]. Thus, the dysregulation of miRNAs or lncRNAs contributes to the aberrant function of MET signaling in glioblastoma.

## Downstream signaling and cross-talk between MET and other molecules in gliomas

In addition to activating mutations in MET and the dysregulation of modulators of this RTK, the activation of downstream signaling and cross-talk between MET and other molecules have also been demonstrated in gliomas.

The downstream signal transduction mediators of HGF/MET signaling in gliomas include Ras/MAPK, PI3K/Akt, and STAT pathways, which mediate a variety of cellular behaviors including proliferation, survival, cell cycle progression, angiogenesis, invasion, migration, stemness, and therapeutic resistance and recurrence in glioblastomas [15–20]. In recent years, mounting evidence has suggested that the interactions between several other signaling pathways and the HGF/MET signaling pathway play a vital role in the pathogenesis of glioblastoma. As is known, Wnt/β-catenin signaling is a key downstream mediator of MET signaling, and both signaling pathways are hyperactive in human gliomas [55]. A further study showed that they both regulate the proliferation, migration and stem cell behavior of glioblastoma cells by increasing the phosphorylation of β-catenin (Y142) and expression of Snail/Slug [56]. Another pathway, the Cox-2/PGE2 axis, can affect most of the hallmarks of cancer [57, 58], and directly activates PGE2-dependent downstream pathways including Ras- MAPK, among others [59]. In gliomas, HGF/MET signaling has been demonstrated to promote tumor growth and migration via the up-regulation of Cox-2 expression and the stimulation of PGE2 release [60]. CD44 is a multifunctional transmembrane glycoprotein receptor of hyaluronan that participates in the development of various solid tumors [61]. Xu et al. first reported that CD44 is a co-stimulator of the MET signaling pathway in glioma cells and attenuated CD44 expression was found to diminish the HGF-induced phosphorylation of Erk1/2 kinase but not that of AKT kinase, suggesting that CD44 preferentially modulates proliferation but not survival signaling pathways activated by HGF growth factors [62]. Moreover, the MET/PKCδ/SRC/STAT3 signaling axis can activate subsequent NOTCH2 signaling, and ultimately leads to increased invasiveness of glioblastoma cells [63]. Chemokine receptors are known to play pivotal roles in the increased migration of many tumors [64]. Esencay et al. revealed that HGF upregulates CXCR4 protein expression which is mediated by NF-kB, and increases the migration ability of glioma cells towards SDF-1a (the ligand of CXCR4) [64]. Moreover, shedding of the invasion-relevant substrate MET via the protease ADAM8 was found to facilitate resistance to TMZ in glioblastoma cells [65]; however, the possible underlying mechanism associated with this soluble MET molecule remains unclear.

Several other molecules and axes associated with HGF/MET signaling have been found to contribute to the stem cell phenotype and aberrant vascularization of glioblastomas. *SOX2* encodes a core transcription factor essential for maintenance of the self-renewal capacity of neural stem cells [66]. In mice lacking Ink4 and Arf tumor suppressors, MET overexpression was found to confer a stem cell phenotype to ionizing radiation-treated glioblastomas via the upregulation of SOX2 [41]. Ganglioside D3 (GD3) is found on the surface of neural stem cells [67]. One study showed that glycolipid GD3 and GD3 synthase are highly expressed in glioma stem cells (GSCs) and play a key role in glioblastoma tumorigenicity through the activation of MET [68]. Recently, Huang et al. provided evidence that MET mediates endothelial plasticity, in which the MET/ETS-1/matrix metalloproteinase-14 (MMP-14) axis controls VE-cadherin degradation, endothelial–mesenchymal transition, and vascular abnormality, driving aberrant vascularization and chemoresistance in glioblastoma [69].

Heat shock protein 90 (HSP90) plays a key role in processes related to protein folding, stabilization, and degradation. In cancer cells, HSP90 is present entirely in multichaperone complexes with high ATPase activity, which are involved in the processing of oncoproteins critical to cancer progression. A study by Miekus et al. demonstrated that the expression of MET receptor is dependent on the presence of HSP90 protein, and thus the HSP90 inhibitor was found to block glioma cell growth and migration through the inhibition of MET receptor expression [70]. In searching for the latest clinical trials on HSP90 inhibitors, there have been fewer advances. In addition, in glioblastomas, there have been no clinical trials testing HSP90 inhibitors to date [71].

HGF/MET signaling also involves cross-talk with EGFR, HER3, and EGFRvIII. EGFRvIII induces the transactivation of JNK2 in glioblastoma cells, and then promotes increased cellular invasion through the stimulation of an HGF/MET signaling circuit [72–74]. Moreover, HGF/MET signaling can induce EGFR and HER-3 activation, leading to enhanced activation of oncogenic signaling in glioblastoma [14, 75].

In human cancers, transforming growth factor-β (TGF-β) signaling can induce tumor-suppressive or tumor-promoting functions depending on the tumor type and the stage of tumor progression [76]. Nevertheless, TGF-β exerts an inhibitory effect on MET phosphorylation and suppresses HGF/MET pathway activity in glioblastoma [77]. Another molecule, FRMD6, is an Ezrin/Radixin/Moesin family protein upstream of the Hippo signaling pathway that controls proliferation, apoptosis, tissue regeneration, and tumorigenesis. A further study confirmed that FRMD6 is downregulated in human glioblastoma cells and tissues and exerts its anti-glioblastoma effect largely through the negative regulation of MET RTK activity [78].

The intricacies of downstream signaling pathways and the cross-talk between MET and other molecules presented in this section indicate the complexity of gliomas; thus, drugs that inhibit single targets could be combined to achieve multiple target inhibition and obtain better treatment results.

## HGF/MET-targeting therapies for glioma

The dysregulation of MET signaling is associated with WHO grades, therapy resistance, recurrence, and poor outcomes for glioma patients [33–35], making this receptor an attractive target for potential treatment. Over the last few decades, therapies comprising antibodies or small-molecule inhibitors targeting MET or HGF have gained extensive attention in numerous preclinical and clinical studies (summarized in Table 2).

**Table 2:** Novel treatment options that are associated with HGF/MET signaling pathway in glioblastoma

Agent	Oral, Intravenous	Molecular type	Mechanisms of Action	Animal model (Subcutaneous, Intracranial)	Clinical trail	Ref.
YYB-101	Intravenous	A humanized monoclonal anti-HGF antibody	Neutralize HGF	Intracranial	NCT02499224 (Phase I)	[79, 80]
Rilotumumab (AMG102)	Intravenous	A neutralizing antibody against HGF	Neutralize HGF	–	NCT01113398 (phase II)	[82, 83]
Onartuzumab	Intravenous	A humanized monovalent monoclonal antibody	Block c-Met receptor	Intracranial (infused intratumorally using osmotic minipumps)	NCT01632228 (phase II)	[84, 85]
Crizotinib	Oral	A tyrosine kinase inhibitor	Target ALK, ROS1, and MET	–	NCT02270034 (phase I) NCT01644773 (phase I)	[86, 87]
Volitinib	Oral	A kinase inhibitor	Inhibit the phosphorylation of c-Met.	Subcutaneous	–	[88]
SGX523	Oral	Small molecule kinase inhibitor	Inhibite c-Met activation	Intracranial	NCT00607399 (phase I), NCT00606879 (phase I)	[89]
INCB28060	Oral	A novel inhibitor of c-MET kinase	Inhibit c-MET enzyme activity	Subcutaneous	–	[75]
Cabozantinib (XL184)	Oral	A molecular kinase inhibitor	Inhibit VEGF receptor 2 (VEGFR2) and MET.	Intracranial	NCT00704288 (phase II)	[92–94]
Altiratinib	Oral	A kinase inhibitor	Inhibit the activation of MET, TIE2, VEGFR2, and tropomyosin receptor kinase family kinases.	Intracranial	–	[95]
CM-118	Oral	A novel lead compound	Selectivity inhibit the phosphorylation of c-Met and ALK.	Intracranial	–	[96]
Brefelamide	–	An aromatic amide that was originally isolated from Dictyostelium cellular slime molds.	Inhibit the secretion of HGF and expression and activation of c-Met.	–	–	[97]
PLB-1001	Oral	A MET kinase inhibitor	High selectively inhibit the activation of Met	Subcutaneous and intracranial	NCT02978261 (Phase I)	[20]

The humanized monoclonal anti-HGF antibody, YYB-101, suppresses tumor growth in vitro and in an orthotopic mouse model of human glioblastoma; it also downregulates important cellular molecular effectors including p-MET, p-Gab1, p-FAK, MMP2, uPA/plasminogen, and Ki-67 [79, 80]. Combination treatment with YYB-101 and TMZ was found to decrease tumor growth and increase OS, compared to the effects of either agent alone, in mice bearing human glioblastoma xenografts [80]. There is also a clinical trial registered for this monoclonal antibody for solid tumors, but with no available results (NCT02499224).

Rilotumumab (AMG102), a neutralizing antibody against HGF, has shown antitumor activity in vitro and in U-87 MG tumor xenograft models as a single agent [81]. Nevertheless, it was not successful in clinical trials against recurrent glioblastoma in 2011 [82]. Another phase II study to evaluate the efficacy and safety of AMG102 and Avastin (bevacizumab) in subjects with recurrent malignant glioma resulted in the conclusion that rilotumumab with bevacizumab does not significantly improve the objective response, as compared to that with bevacizumab alone, and that toxicity might preclude the use of rilotumumab in combination with bevacizumab regimens [83].

Onartuzumab, a humanized monovalent monoclonal anti-MET antibody, resulted in the inhibition of glioblastoma growth in preclinical testing [84]. However, in a phase II clinical trial for recurrent glioblastoma, this agent plus bevacizumab, versus a placebo plus bevacizumab, showed no evidence of further clinical benefit [85].

Crizotinib, an available ATP competitive selective inhibitor, was originally developed as an inhibitor of MET, but it also inhibits structurally-related tyrosine kinases such as ALK and the ROS proto-oncogene 1 (ROS1) [86]. It effectively inhibits the proliferation and survival of MET-positive GSCs, rather than MET-negative GSCs, and apparently prolongs the survival of mice bearing MET-positive GSCs [87]. Nevertheless, to date, there have been only two ongoing phase I clinical trials in recent years to evaluate the safety and activity of crizotinib with TMZ and radiotherapy for newly diagnosed glioblastoma or to evaluate the tolerable dose of crizotinib and dasatinib in pediatric patients with diffuse pontine glioma and high-grade glioma (NCT02270034, NCT01644773).

Volitinib is a highly selective small molecule, ATP competitive MET kinase inhibitor that is being investigated as a monotherapy for MET-amplified cancers such as gastric and lung cancer. However, for glioblastoma, there has only been one preclinical study that has demonstrated good anti-tumor activities using a human xenograft model in athymic nude mice [88]. No further studies using this agent for gliomas have been registered as clinical trials.

The small molecule inhibitor, SGX523, potently inhibits MET activation and MET-dependent signaling in glioma cells and inhibits proliferation, cell cycle progression, migration, invasion, and in vivo tumor growth [89]. However, the two clinical trials registered for this agent for the treatment of solid tumors were terminated without available results (NCT00607399, NCT00606879).

INCB28060 is a potent and selective inhibitor of MET kinase and shows strong anti-tumor activity in MET-dependent mouse tumor models [75]. However, there have still been no clinical trials testing this agent.

Cabozantinib (XL184), a potent inhibitor targeting MET and VEGFR2, exerts anti-angiogenic, anti-proliferative, and anti-invasive effects in animal xenograft models [90, 91]. A preclinical study showed that cabozantinib prolongs the survival of mice bearing orthotopic E98-xenografts by inhibiting tumor proliferation and invasion [92]. The MET pathway has been implicated in resistance to bevacizumab therapy and the pathogenesis of glioblastoma. However, cabozantinib treatment showed only modest clinical activity for this patient population (NCT00704288) [93]. For recurrent glioblastoma naive to anti-angiogenic therapy, cabozantinib showed evidence of clinical activity in these patients, although the predefined statistical target for success was not met (NCT00704288) [94]. Although 5 years have already passed, there has been no phase III clinical trials on this agent for gliomas.

Altiratinib is a novel inhibitor of MET, TIE2, VEGFR2, and tropomyosin receptor family kinases. A study conducted by Piao et al. demonstrated that in multiple xenograft mouse models, altiratinib combined with bevacizumab dramatically reduced tumor volume and prolonged OS compared to those with bevacizumab alone [95]. However, for this agent, no clinical trials have been registered in ClinicalTrials. gov.

CM-118 is a novel lead compound against both ALK and MET with high specificity, as compared to that for 90 human kinases. It selectively inhibits the proliferation of MET-addicted U87MG cells in vitro and was found to elicit the tumor regression of U87MG xenografts in mice after oral administration at a dose of 60 mg/kg [96]. Although this drug worked well in this previous study, no further research has since been reported regarding this compound.

Brefelamide is an aromatic amide that was originally isolated from *Dictyostelium* cellular slime molds. It was found to inhibit the growth of human astrocytoma cells through the reduced expression and activation of MET and reduced the secretion of HGF [97]. Nevertheless, no further study has been reported for this agent.

PLB-1001 is a highly selective, efficient, and blood-brain-barrier (BBB)-permeable MET kinase inhibitor. It was previously characterized and demonstrated effective suppression of MET-induced glioma progression in cell lines and xenografts; further, in an open-label phase I clinical trial, the safety and efficacy of PLB-1001 for the treatment of patients with a ZM fusion and/or METex14 was shown [20].

Since there have been no phase III clinical trials for these therapies with respect to gliomas, it is of great importance to identify the patient subgroups most likely to benefit from these targeted therapies and conduct further studies to assess the penetration of these agents through the BBB. Moreover, with respect to the heterogeneity of gliomas, combination therapies should be mainly considered.

## Current situation regarding targeted therapy in clinical practice

As is known, aberrant RTK signaling is a key driver of tumorigenesis and resistance to treatment in glioblastoma [14]. Although EGFR mutations, amplification, and overexpression are common in glioblastoma and gefitinib is well tolerated in patients with malignant gliomas, treatment is not associated with significant improvements in OS or PFS compared to that in the historical control population [13]. Of note, inhibition of EGFR induces a MET-driven stem cell population in glioblastoma [98]. Joo et al. identified a distinct fraction of cells expressing a high level of MET and co-expressing GSC markers in human primary glioblastoma specimens, which were found to be highly clonogenic, tumorigenic, and resistant to radiation [99]. EGFRamp tumors exhibit erlotinib resistance and respond to a combination of MET and EGFR inhibitors, which was demonstrated through the use of intracranial xenograft glioma models [100]. Thus, the application of new combined therapies for clinical treatment deserves further attention.

In 2009, the U.S. Food and Drug Administration accelerated the approval of bevacizumab, a humanized monoclonal antibody against VEGF, as a single agent, based on its therapeutic benefit in recurrent glioblastoma patients [15]. Subsequently, its use in the frontline setting for newly diagnosed glioblastoma had been evaluated; however, compared to that with TMZ, it only prolongs PFS but not OS (median PFS: 10.7 months vs. 7.3 months; median OS, 15.7 and 16.1 months) [30]. Further, the inhibition of VEGF signaling leads to a proinvasive phenotype in a subset of glioblastoma patients and in mouse models of glioblastoma treated with bevacizumab [82, 101]. It was later found that VEGF directly and negatively regulates tumor cell invasion through the enhanced recruitment of protein tyrosine phosphatase 1B (PTP1B) to a MET/VEGFR2 heterocomplex, thereby suppressing HGF-dependent MET phosphorylation and tumor cell migration [15]. Bevacizumab-resistant glioblastomas present with increased MET phosphorylation and increased phosphorylation of MET-activated focal adhesion kinase and STAT3, which suggests a role for MET in features associated with anti-angiogenic therapy resistance both in vitro and in vivo [91]. Onartuzumab, a humanized monoclonal anti-MET antibody, inhibited glioblastoma growth in a preclinical testing [84]; however, the combination treatment of onartuzumab with bevacizumab showed no clinical benefit compared to that with bevacizumab plus placebo [85].

Collectively, EGFR- and VEGF-targeted therapies seem to contribute little to the treatment of gliomas in current situations. Further, one paper reported that the majority of targeted molecular drugs evaluated for malignant gliomas result in response rates of only 10 to 15% or less and no prolongation of survival [102]. Thus, there is a long way to go regarding the treatment of glioblastoma.

## Discussion

Among all gliomas, glioblastomas, regardless of whether they are primary or secondary, are the most devastating and intractable disease and are associated with dismal outcomes. Standard treatment for glioblastoma involves maximum surgical resection followed by the Stupp regimen consisting of fractionated radiotherapy plus concomitant TMZ chemotherapy, as well as 6–12 cycles of adjuvant TMZ chemotherapy. Despite this aggressive therapy, the median OS is 14.5–16.6 months, and the 2-year and 5-year OS rates are 27.2 and 5.5%, respectively [1, 2, 103]. As such, there has been considerable interest in recent years in the application of targeted approaches for glioblastoma patients.

**Fig. 2:**
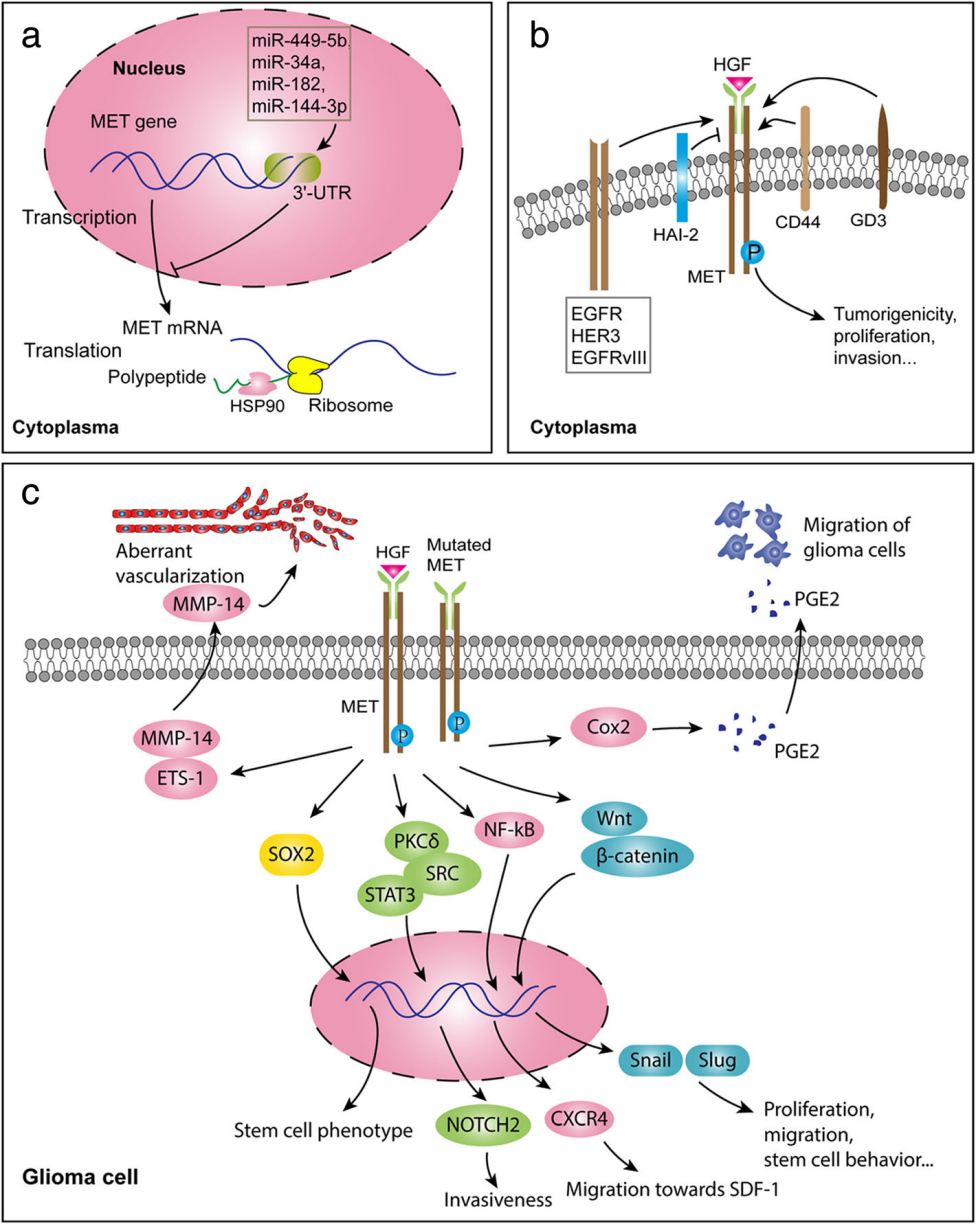
The regulation of MET expression and activation, and representative signal pathways associated with MET signaling. A. MiR-449-5b, miR-34a, miR-182, and miR-144-3p specifically bind the MET 3′-UTR region and inhibit MET transcription. Downregulation of these miRNAs upregulates the expression levels of MET. HSP90 facilitates the translation and modification of MET protein. B. Several other membrane proteins participate in the activation of MET; HAI-2 inhibits HGF-induced phosphorylation of MET, whereas CD44, GD3, and some other RTKs (EGFR, HER3, EGFRvIII) promote the phosphylation of MET, which ultimately promotes the tumorigenicity, proliferation, and invasion of glioma cells. C. MET signaling is associated with downstream signaling such as Wnt/β-catenin/Snail/Slug, NF-kB/CXCR4/SDF-1, PKCδ/SRC/STAT3/NOTCH2, Cox2/PGE2, ETS-1/MMP-14, and the stem cell transcription factor SOX2, all of which facilitate proliferation, migration, invasion, stem cell behavior, and aberrant vascularization in gliomas

Due to the high level of heterogeneity, glioblastomas usually contain a mixture of cells with the amplification and activation of multiple RTKs. Therefore, targeting a single RTK might not be sufficient to inhibit glioblastoma [104]. It has been demonstrated that MET and its ligand HGF play a critical role in the proliferation, survival, migration, invasion, angiogenesis, stem cell characteristics, and therapeutic resistance and recurrence of glioblastomas [15–20]. As presented in this review, the dysregulation of miRNAs (miR449-5b, miR-34a, miR-182, and miR-144-3p) contributes to over-transcription of the *MET* gene, and HSP90 is essential for the translation and modification of the MET protein (Fig. 2a). Moreover, cross-talk between MET and other membrane molecules and signaling pathways plays essential roles in the activation of MET signaling and functions importantly in the malignant progression of gliomas (Fig. 2b, c). In light of HGF/MET-targeting therapies, the disappointing results of those preclinical studies with respect to their translation into clinical studies might result from the limitations of animal models to forecast efficacy for patients, as well as substantial differences between intracranial glioblastoma xenograft models and human intracranial glioblastomas. To date, the inhibition of multiple targets has gained considerable interest to combat drug resistance in glioblastoma. However, understanding the molecular mechanisms underlying cross-talk between signaling pathways and predicting the responses of cancer cells to targeted interventions remain challenging, and this depends not only on the essential knowledge of the molecular features of drugs and targets, but also the proper selection of the patient population likely to respond favorably to specific treatments.

Clearly, it remains insufficient for the advances achieved in the treatment studies for malignant gliomas as they rapidly develop resistance. As we enter the era of targeted therapy and personalized medicine, the development of biomarkers to help select the most appropriate patient population for a specific therapy is key. Rigorous preclinical testing is needed to identify combinations of drugs and targets that are most likely to be effective and tolerated. Although the initial results for HGF/MET signaling-targeted therapies seem disappointing, molecular targeted therapeutic agents hold tremendous promise. Therefore, it is expected that a further understanding of drug modifications, the selection of targeted sites, the tumor immune microenvironment, the complex network of interactions between different tumor cell populations, and the penetration of proper drugs across the BBB will provide us with more thorough insights to find more effective treatment strategies. We should remain optimistic that the ultimate goal of identifying targeted molecular therapies with robust anti-tumor efficacy will be realized for gliomas as it has been for lung cancer and leukemia.

## Conclusions

This review describes the role of MET signaling in gliomas, among which glioblastoma presents a major challenge with limited treatment options and poor prognosis. MET and its ligand hepatocyte growth factor (HGF) play a critical role in the proliferation, survival, migration, invasion, angiogenesis, stem cell characteristics, and therapeutic resistance and recurrence of glioblastomas. The progress made in understanding of MET signaling in glioma and advances in therapies targeting HGF/MET molecules for glioma patients in recent 30 years were highlighted, in addition to studies on the expression and mutation status of MET. Our review makes a significant contribution to the latest concepts related to MET signaling and targeted therapies for glioma, as combined targeted therapy for this pathway and associated molecules remains an attractive strategy for the treatment of this disease.

## Data Availability

Not applicable.

## Abbreviations

ATRX: α-thalassemia/mental retardation syndrome X-linked gene
CGH: Comparative genomic hybridization
FISH: Fluorescence in situ hybridization
GBM: Glioblastoma multiforme
GSCs: Glioma stem cells
HAI-2: Hepatocyte growth factor activator inhibitor type 2
HSP90: Heat shock protein 90
IDH1/2: isocitrate dehydrogenase 1/2
MAPK: Mitogen-activated protein kinase
MET: Mesenchymal-epithelial transition factor
METex14: MET-exon 14-skipping
MGMT: O6-methylguanine-DNA methyltransferase
MMP-14: Matrix metalloproteinase-14
OS: Overall survival
PDGFRα: Platelet derived growth factor receptor α.
PFS: Progression-free survival
PI3K: Phosphoinositide 3-kinase
PTEN: Phosphatase and tensin homolog
RTK: Receptor tyrosine kinase
SDF-1a: Stromal cell-derived factor-1α
TERT: Telomerase reverse transcriptase
TMZ: Temozolomide
WHO: World Health Organization
